# InGaAs/InAlAs single photon avalanche diode for 1550 nm photons

**DOI:** 10.1098/rsos.150584

**Published:** 2016-03-16

**Authors:** Xiao Meng, Shiyu Xie, Xinxin Zhou, Niccolò Calandri, Mirko Sanzaro, Alberto Tosi, Chee Hing Tan, Jo Shien Ng

**Affiliations:** 1 Department of Electronic and Electrical Engineering, University of Sheffield, Sheffield S1 3JD, UK; 2 Dipartimento di Elettronica, Informazione e Bioingegneria, Politecnico di Milano, Milano 20133, Italy

**Keywords:** single photon avalanche diode, photon counting, fibre-optic telecommunication

## Abstract

A single photon avalanche diode (SPAD) with an InGaAs absorption region, and an InAlAs avalanche region was designed and demonstrated to detect 1550 nm wavelength photons. The characterization included leakage current, dark count rate and single photon detection efficiency as functions of temperature from 210 to 294 K. The SPAD exhibited good temperature stability, with breakdown voltage dependence of approximately 45 mV K^−1^. Operating at 210 K and in a gated mode, the SPAD achieved a photon detection probability of 26% at 1550 nm with a dark count rate of 1 × 10^8^ Hz. The time response of the SPAD showed decreasing timing jitter (full width at half maximum) with increasing overbias voltage, with 70 ps being the smallest timing jitter measured.

## 1. Introduction

Applications that rely on photon counting are increasing in number as well as in their significance. Among the important examples are quantum key distribution [1], eye-safe three-dimensional imaging [2], optical time domain reflectometry [3] and CMOS circuit testing [4]. For most applications, single photon avalanche diodes (SPADs), a variant of avalanche photodiodes (APDs), remain the detector of choice, despite superconducting single photon detectors (SSPDs) [5] achieving high detection efficiency and low dark counts. This is primarily owing to practical consideration, because SPADs have a far more moderate cooling requirement (multi-stage thermoelectric cooler) compared with SSPDs (operation temperature below 4 K).

The vast majority of SPADs being used have a planar structure similar to those of fibre-optic telecommunication InGaAs/InP APDs [6,7], that use an InGaAs absorption layer and an InP multiplication layer. They are optimized for detection of photons at the wavelength of 1550 nm. The first custom-designed InGaAs/InP SPADs gave 10% photon detection probability (*PDP*) and 2 × 10^5^ Hz dark count rate (*DCR*) at 200 K [8]. Since then much better performance is available from these SPADs, with *PDP* reports of 38% at 225 K [9] and even up to 55% at room temperature [10]. In addition to improving the current InGaAs/InP SPADs, it is worth considering if other semiconductor materials have greater potential or other advantages as the avalanche region of SPADs designed for 1550 nm wavelength.

Simulations [11] have shown that, for a given *DCR*, SPADs using InAlAs instead of InP as avalanche material achieve higher *PDP* because of higher avalanche breakdown probability (proportional to *PDP*) in InAlAs. Moreover, for a given avalanche region width, avalanche breakdown voltage of InAlAs is less sensitive to temperature than that of InP [12], offering greater flexibility in the SPAD operation temperature. Despite these advantages, there has been limited research on SPADs with InAlAs avalanche regions [13–15]. Recently, we reported an InGaAs/InAlAs SPAD with *PDP* of 21% at 260 K [16]. However, the device exhibited excessive *DCR* that hardly drops with temperature, a characteristic attributed to tunnelling current originating from the InAlAs avalanche region. In this work, we designed, fabricated and characterized an InGaAs/InAlAs SPAD with an improved structure. *PDP* of 26% and *DCR* of 1 × 10^8^ Hz were obtained when the SPAD was cooled to 210 K.

## 2. Experimental details

**Figure 1.**
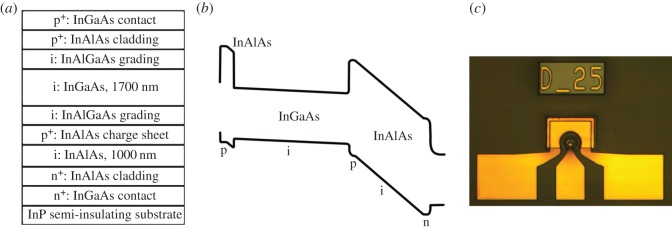
(*a*) Structure details of the InGaAs/InAlAs SPAD. (*b*) Energy band diagram of the SPAD under reverse bias. (*c*) Photograph of the mesa InGaAs/InAlAs SPAD (25 µm active area diameter) and its bond pad.

Our InGaAs/InAlAs SPAD was grown by molecular beam epitaxy on a semi-insulating InP substrate at the EPSRC National Centre for III–V Technologies at the University of Sheffield. As shown in figure 1, the wafer consisted of a 1700 nm InGaAs absorption region and a 1000 nm InAlAs avalanche region. Compared with the design used in [16], the wafer has thicker absorption and avalanche regions, increasing the photon absorption efficiency and reducing the tunnelling current from InAlAs, respectively. A thin InAlAs charge sheet layer (doping density >1 × 10^17^ cm^−3^) was used to achieve a large difference in the electric fields in absorption region and avalanche region. InAlGaAs layers with intermediate bandgaps were included for bandgap grading at InGaAs/InAlAs heterojunctions.

Top-illuminated mesa devices with diameters of 10–50 µm were fabricated from the wafer using standard photolithography and wet chemical etching with a solution of sulfuric acid : hydrogen peroxide : deionized water (ratio of 1 : 8 : 80). The p-contacts and n-contacts were formed by annealed metals of Ti/Pt/Au (10/30/200 nm). The devices were passivated by negative photoresist SU8. Bond pads to the p- and n-contacts were formed by depositing Ti/Au (10/500 nm). No anti-reflection coating was applied. Results shown in §3 were obtained from the 25 µm diameter SPADs, which is the typical size for commercial SPADs at 1550 nm wavelength.

Current–voltage (I–V) measurements of the device at temperatures from 210 to 294 K were performed using a Janis probe station connected to a source–measure unit. Photon counting characterization on our SPAD was carried out using the set-up described in [16], which also used the Janis probe station. The SPAD was operated in gated mode, using AC electrical pulses with an on-time of *t*_on_ = 1.2 ns (full width at half maximum, FWHM) and 20 V amplitude superimposed on a varying DC bias (below breakdown voltage). Repetition frequencies of the electrical pulses, *f*, were 100 and 10 kHz for operating temperatures of 210–294 K and 150–170 K, respectively. The electrical pulses therefore had a duty cycle ratio of *δ* = *t*_on_ × *f*. For *DCR* measurements, the gated mode tests yielded *DCR* = *C_*d*_*/*δ*, where *C_*d*_* is the measured count rate. Laser pulses (1550 nm wavelength and 20 ps FWHM) at the same frequency as the electrical pulses were synchronized with the AC electrical pulses by adjusting the delay between them. The laser pulses were attenuated to single photon level (average number of photons per pulse, *N*, was 0.3) using a variable optical attenuator. Assuming Poissonian statistics for the arriving photons, *PDP* is expressed as [17] $PDP=(1/N)\ln((1-P_{d})/(1-P_{t})),$ where *P_*t*_* and *P_*d*_* are the measured probability of having an avalanche event for an electrical pulse when the SPAD is illuminated with the photon pulses and in the dark, respectively. *P_*d*_* was obtained experimentally from *P_*d*_* = 1 − exp(−*C_*d*_*/*δ*). *P_*t*_* was obtained similarly with the total count rate taken when the SPAD was illuminated (i.e. with photon counts and dark counts).

Another important SPAD parameter is the temporal response, typically measured by the FWHM of the arrival time distribution of photons emitted by a very sharp pulsed laser. We employed a 1550 nm pulsed laser (FWHM less than 20 ps) focused into a 5 µm spot at the centre of the active area of the SPAD, which was kept at 210 K. The laser pulses had power at a lower level than that used for the *PDP* measurements (i.e. less than 0.3 photons per pulse), so that detection probability is less than 5%, which guarantees negligible distortion in the optical waveforms reconstructed with the time-correlated single photon counting technique. The power was kept constant for all temporal measurements. The SPAD was again operated in gated mode, with AC electrical pulses (10 ns pulse width, 10 kHz repetition rate and varying pulse amplitude) and DC reverse bias 0.5 V below its breakdown voltage.

## 3. Results

**Figure 2.**
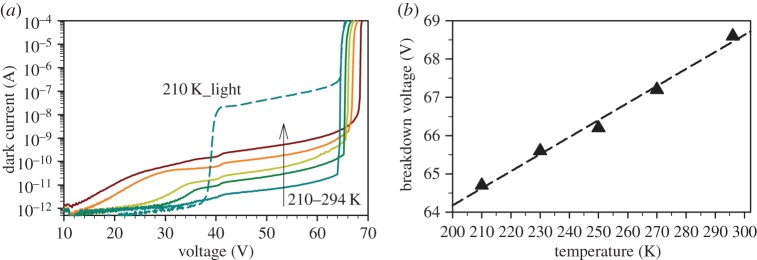
(*a*) Dark currents (solid lines) of a 25 µm diameter InGaAs/InAlAs SPAD at 210, 230, 250, 270 and 294 K (bottom to top). Photocurrent (dashed line) at 210 K when the SPAD is flood-illuminated with 1550 nm laser with optical power of approximately 40 nW. (*b*) Breakdown voltage versus temperature data (symbols) and linear fitting (line).

Figure 2*a* shows the typical dark I–V data of a 25 µm diameter SPAD at temperatures from 210 to 294 K as well as the photocurrent at 210 K when the SPAD was flood-illuminated with a 1550 nm continuous-wave laser with approximately 40 nW power. The dark current at 95% of breakdown voltage was 17 pA and 2.6 nA at 210 and 294 K, respectively. The photocurrent data indicate a punch-through voltage, the minimum voltage to fully deplete the entire SPAD structure, of approximately 42 V. At room temperature, the responsivity of the SPAD at punch-through voltage is 0.7 A/W, giving an external quantum efficiency of 56%. This gives an upper limit of 56% for *PDP* (because probability of photo-generated carriers to reach the avalanche region and probability of avalanche breakdown do not exceed unity).

Plotting breakdown voltage (estimated as the voltage at which current reaches 10 µA) from the dark I–V data versus temperature in figure 2*b*, its temperature coefficient, *C*_bd_, was found to be 45 mV K^−1^. This value is close to 50 mV K^−1^, the estimate using equations (1) and (2) from [12], and about half of that obtained from InGaAs/InP SPADs (approx. 100 mV K^−1^ [10]). This small *C*_bd_ ensures that the breakdown voltage is always higher than the punch-through voltage, over the wide temperature range studied in this work. In terms of SPAD design, the breakdown voltage could afford to be closer to the punch-through voltage, to further reduce the electric field in the InGaAs absorption region.

**Figure 3.**
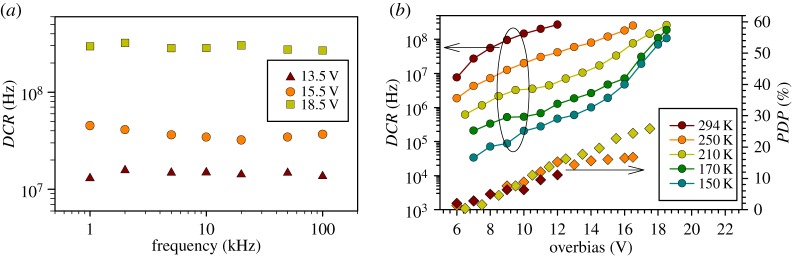
(*a*) *DCR* versus repetition frequency of the 1.2 ns pulses at 210 K as a function of overbias, and (*b*) *DCR* and *PDP* versus overbias for temperatures ranging from 150 to 294 K.

Selection of the gate frequency of the electrical pulses for subsequent characterization involved measurements of *DCR* versus gate frequency ranging from 1 to 100 kHz (maximum operating frequency of the pulser), as a function of overbias (i.e. the difference between the SPAD bias and its breakdown voltage). The data obtained from our device at 210 K, with overbias up to 18.5 V are shown in figure 3*a*. The data were not dependent on frequency up to 100 kHz, so afterpulsing effect was negligible in these conditions. This is expected, because the narrow AC pulses used limit the total number of carriers generated during an avalanche breakdown event, and hence the number of trapped carriers, which are responsible for afterpulsing effect. Additionally, the low duty cycle of the gate waveform (given by the short gate width of 1.2 ns) contributes to few afterpulses per gate period.

*DCR* and *PDP* versus overbias of the device are plotted as functions of temperature in figure 3*b*. At 294 K, the highest *PDP* achieved is 17%. With 12 V overbias, cooling the SPAD from 294 to 210 K reduces the *DCR* by nearly two orders of magnitude, indicating that the *DCR* at these temperatures is not dominated by tunnelling currents from avalanche region, which was the case for SPADs in reference [16]. The lower *DCR* at 210 K allows higher overbias voltage to be applied, yielding *PDP* as high as 26% at 1550 nm. Adding an anti-reflection coating is expected to increase this value to approximately 37%.

Measurements of *DCR* versus *PDP* at 210 K were repeated on another four devices from the same piece of sample. For a given *PDP*, the variation in *DCR* was well within an order of magnitude. It is also informative to assess the performance of the SPADs by evaluating signal-to-noise ratio, SNR, defined as

3.1 $$\mathrm{SNR}=\frac{S}{\sqrt{S+N}}=\frac{PDP\times N}{\sqrt{T_{\mathrm{int}}}\times \sqrt{(PDP\times N)+DCR}},$$

where *T*_int_ is the integration time of the instrument. For approximately 0.3 photons per pulse, if *T*_int_ = *t*_on_ = 1.2 ns (the overbias pulse width), then the highest SNR obtained was approximately 0.59 when the SPAD was operated at 210 K. The corresponding *PDP* and *DCR* were 13% and 4 × 10^6^ Hz, respectively.

**Figure 4.**
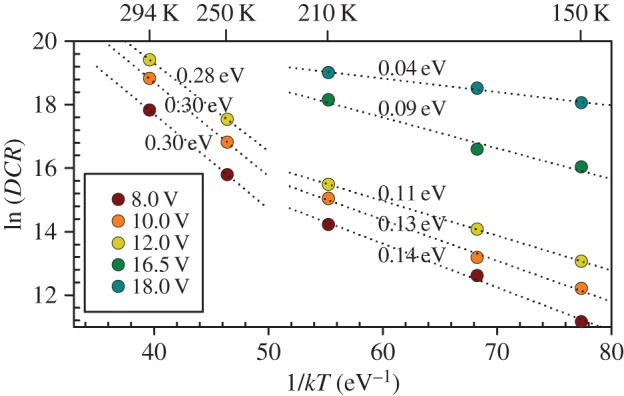
Data of ln(*DCR*) versus 1/*kT* as a function of overbias. The corresponding activation energies for lower and higher temperatures are reported for each curve.

Possible origins of the dark counts were investigated through deduction of activation energy. Activation energy for the *DCR* at two temperature ranges, 150–210 K and 250–294 K, were obtained from linear fittings to ln(*DCR*) versus 1/*kT* characteristics, as shown in figure 4. Activation energies of approximately 0.1 and 0.3 eV were deduced for the two temperature ranges, respectively, for overbias up to 12 V (corresponding to *PDP* up to approx. 15%). An activation energy of 0.3 eV for the higher temperatures (250–294 K) is consistent with those reported on InGaAs/InP SPADs at similar temperature range [17–19] (0.3–0.5 eV), which are attributed to thermal generation current in the InGaAs absorption layer. At lower temperatures (150–210 K), the lower activation energy indicates that the dominant origin of dark counts is likely to be tunnelling-related mechanism [15], which is less temperature-dependent and more prominent at low temperatures [20]. Furthermore, the increase in activation energy with temperature is in line with other works [18,19].

Observing figure 4, as overbias increases beyond 12 V, the activation energy decreases down to approximately 0.04 eV at the highest overbias used. This is likely to be caused by tunnelling-related current growing in significance with overbias. This is similar to the observation made by Karve *et al*., who found their InGaAs/InAlAs SPADs with high band-to-band tunnelling current from the InAlAs avalanche region exhibiting a small activation energy (0.12–0.15 eV) even at high temperatures (up to 280 K) [13]. However, it does not necessarily mean that the same dominant dark count mechanism applies to our SPAD at high overbias, because other tunnelling-related mechanisms, such as trap-assisted tunnelling, can also give rise to very small activation energy.

**Figure 5.**
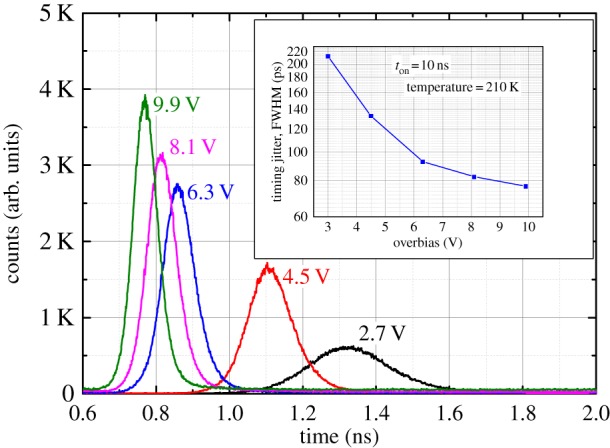
Photon timing jitter of an InGaAs/InAlAs SPAD at 210 K at a few overbias voltages. The inset shows the FWHM of each curve.

The time response of the SPAD at different overbias is shown in figure 5. The response is clean, and timing jitter is good at high overbias voltage, achieving 70 ps at approximately 10 V overbias, corresponding to a *PDP* of 10%.

## 4. Comparison with other reports

**Figure 6.**
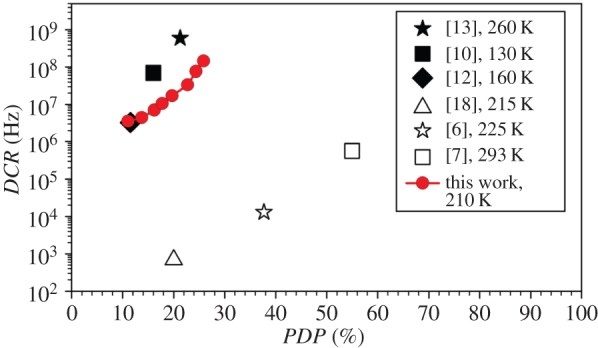
Comparison of *DCR* versus *PDP* results of InGaAs/InAlAs (filled symbols) and InGaAs/InP (open symbols) SPADs from various works.

*DCR* and *PDP* of this work are compared with results from various reports on InGaAs/InAlAs SPADs [13,15,16] and InGaAs/InP SPADs [9,10,21] in figure 6. Compared with our previous InGaAs/InAlAs SPAD [16], this work demonstrates reduced *DCR* (approx. two orders of magnitude) for a given *PDP* and improved maximum *PDP* (21–26%). The increase in maximum *PDP* is attributed to the thicker absorption region (1700 nm instead of 600 nm) that gives higher absorption efficiency and to the thicker multiplication region that gives higher avalanche triggering efficiency. The thicker avalanche region, with its reduced band-to-band tunnelling current, is responsible for the reduction in *DCR*.

In comparison with other works, this one reports the highest *PDP* among all InGaAs/InAlAs SPADs, though it is still lower than the impressive *PDP* from recent InGaAs/InP SPADs [9,10]. However, the *DCR* values remain much higher than those of InGaAs/InP SPADs. This is in part related to the quality of InGaAs layer in our device. Based on measurements on large diodes with diameters of 100–400 µm, we estimated bulk dark current density at punch-through to be 19 µA cm^−2^, at least two orders of magnitude higher than typical values from commercially available InGaAs photodiodes at low reverse bias (approx. 0.1 µA cm^−2^). This is also supported by the activation energy that indicates the dominance of thermal generated carriers for the InGaAs absorption layer. Reducing the thermally generated carriers from the InGaAs layer should therefore lower the *DCR* significantly.

Finally, temporal response of these devices to pulsed laser focused at the centre of the active area is comparable to state-of-the-art InP-based SPADs (less than 90 ps) [9,21]. As the overbias increases, the breakdown probability increases, leading to smaller timing jitter as expected [22]. These data are the first comprehensive characterization of InGaAs/InAlAs SPADs.

## 5. Conclusion

An InGaAs/InAlAs SPAD with relatively thick InAlAs avalanche layer (1.0 µm cf. 0.2 µm in previous work) was demonstrated. Its temperature stability is good with approximately 45 mV K^−1^ temperature coefficient for the avalanche breakdown voltage. Using gated mode operation, the SPAD exhibited 26% photon detection probability and 1 × 10^8^ Hz dark count rate at 210 K. The best SNR achieved for 1.2 ns integration time and 0.3 average photons per pulse was approximately 0.59, also at 210 K. Timing jitter of the SPAD (70 ps) was found to be comparable to state-of-the-art InP-based SPADs. Temperature dependence of dark count rate indicated different mechanisms being dominant at different overbias ranges. At the high overbias needed to achieve high detection efficiency, the dark counts are mainly owing to tunnelling-related mechanisms.

## Data accessibility

The data reported in this article have been uploaded on the Dryad digital repository (http://dx.doi.org/10.5061/dryad.v8hk0).
